# Supplementary material for: Reevaluating claims of ecological speciation in Halichoeres bivittatus
Source: Ecol Evol. 2021 Jul 26;11(16):11449–56. doi: 10.1002/ece3.7936 (PMC8366890; doi:10.1002/ece3.7936)
Supplement: Supplementary file 1 — Supplementary Material [file ECE3-11-11449-s002.docx]

Supplement S1. Figures visualizing the p values for pairwise comparisons of populations and habitat types for *Halichoeres bivittatus* in the Florida Keys.

**
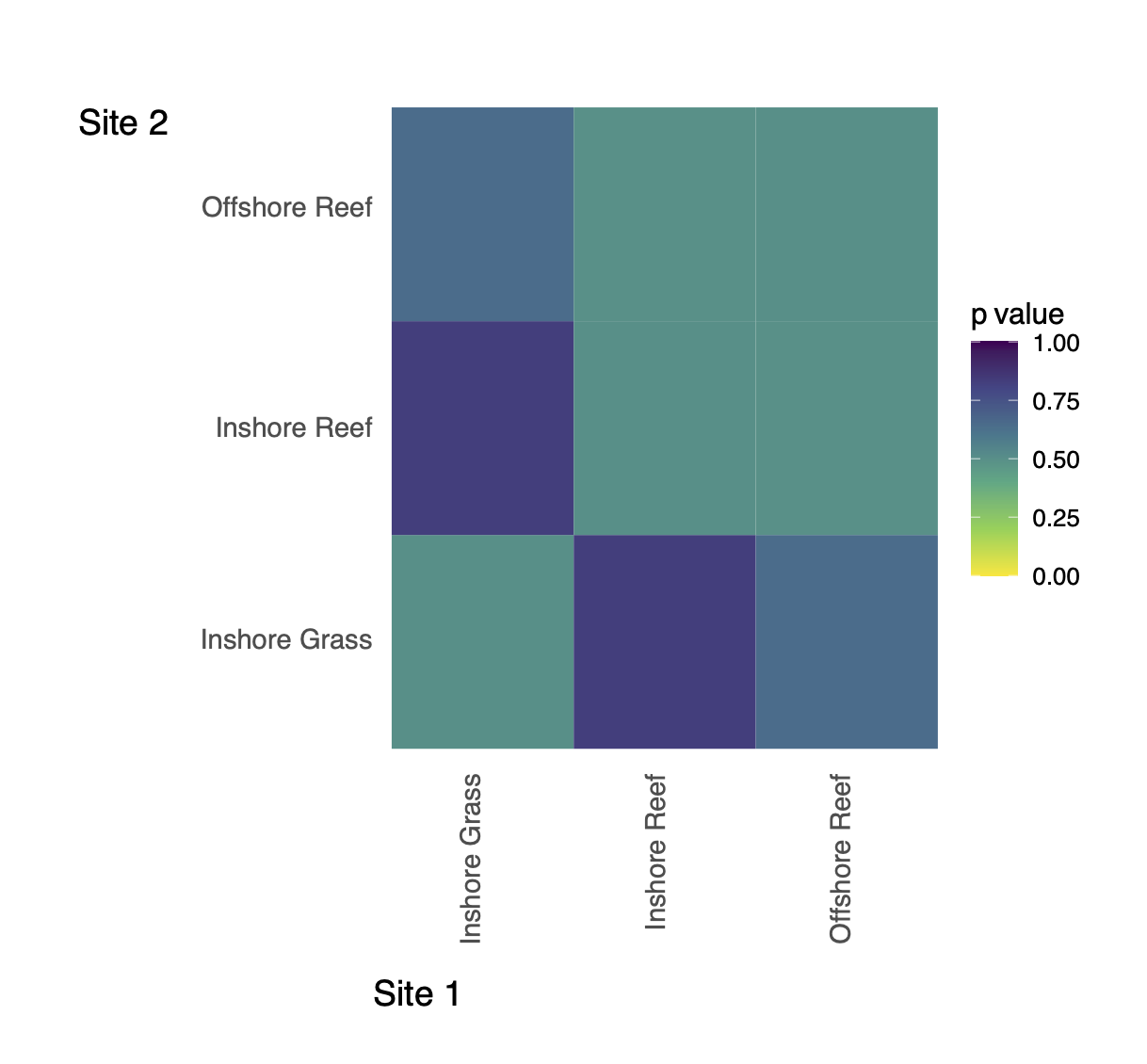
**

Figure S1.1. Significance of Fst values from permutation tests, sites grouped by habitat type. Colors represent p values based on 1000 permutations.


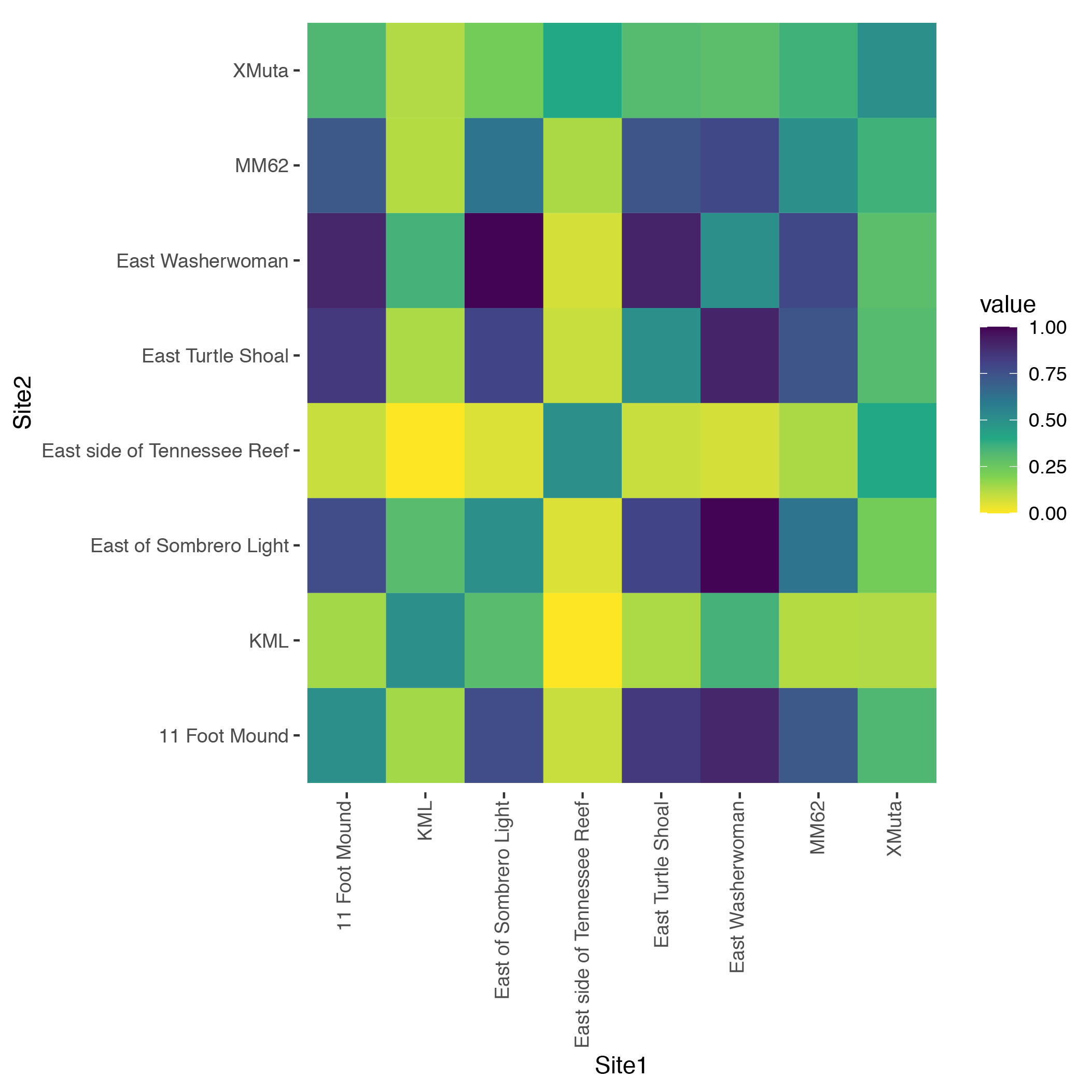


Figure S1.2. Statistical significance of Fst values from permutation tests, comparing each site individually. Colors represent p values based on 1000 permutations. Only one comparison (East Side of Tennessee Reef vs. KML) was statistically significant at p < .05.

**Literature Cited**

[Benson, Dennis A., Mark Cavanaugh, Karen Clark, Ilene Karsch-Mizrachi, David J. Lipman, James Ostell, and Eric W. Sayers. 2013. “GenBank.” *Nucleic Acids Research* 41 (Database issue): D36–42.](http://paperpile.com/b/uQmu7a/scdr)

[Bernardi, Giacomo, Ricardo Beldade, Sally J. Holbrook, and Russell J. Schmitt. 2012. “Full-Sibs in Cohorts of Newly Settled Coral Reef Fishes.” *PloS One* 7 (9): e44953.](http://paperpile.com/b/uQmu7a/L008)

[Bird, Christopher E., Brenden S. Holland, Brian W. Bowen, and Robert J. Toonen. 2011. “Diversification of Sympatric Broadcast-Spawning Limpets (Cellana Spp.) within the Hawaiian Archipelago.” *Molecular Ecology* 20 (10): 2128–41.](http://paperpile.com/b/uQmu7a/LLh8)

[Bowen, Brian W., Luiz A. Rocha, Robert J. Toonen, Stephen A. Karl, and ToBo Laboratory. 2013. “The Origins of Tropical Marine Biodiversity.” *Trends in Ecology & Evolution* 28 (6): 359–66.](http://paperpile.com/b/uQmu7a/WpsQ)

[Chenuil, Anne, Thomas Saucède, Lenaïg G. Hemery, Marc Eléaume, Jean-Pierre Féral, Nadia Améziane, Bruno David, Guillaume Lecointre, and Charlotte Havermans. 2018. “Understanding Processes at the Origin of Species Flocks with a Focus on the Marine Antarctic Fauna.” *Biological Reviews of the Cambridge Philosophical Society* 93 (1): 481–504.](http://paperpile.com/b/uQmu7a/e2OZ)

[Choat, John H., Oya S. Klanten, Lynne Van Herwerden, D. Ross Robertson, and Kendall D. Clements. 2012. “Patterns and Processes in the Evolutionary History of Parrotfishes (Family Labridae).” *Biological Journal of the Linnean Society*. https://doi.org/](http://paperpile.com/b/uQmu7a/gooc)[10.1111/j.1095-8312.2012.01959.x](http://dx.doi.org/10.1111/j.1095-8312.2012.01959.x)[.](http://paperpile.com/b/uQmu7a/gooc)

[Cowman, Peter F., Valeriano Parravicini, Michel Kulbicki, and Sergio R. Floeter. 2017. “The Biogeography of Tropical Reef Fishes: Endemism and Provinciality through Time.” *Biological Reviews of the Cambridge Philosophical Society* 92 (4): 2112–30.](http://paperpile.com/b/uQmu7a/QmXz)

[Ekimova, Irina, Ángel Valdés, Anton Chichvarkhin, Tatiana Antokhina, Tabitha Lindsay, and Dimitry Schepetov. 2019. “Diet-Driven Ecological Radiation and Allopatric Speciation Result in High Species Diversity in a Temperate-Cold Water Marine Genus Dendronotus (Gastropoda: Nudibranchia).” *Molecular Phylogenetics and Evolution* 141 (December): 106609.](http://paperpile.com/b/uQmu7a/kfTH)

[Faria, Rui, Kerstin Johannesson, and Sean Stankowski. 2021. “Speciation in Marine Environments: Diving under the Surface.” *Journal of Evolutionary Biology* 34 (1): 4–15.](http://paperpile.com/b/uQmu7a/qWfU)

[Gaboriau, Théo, Fabien Leprieur, David Mouillot, and Nicolas Hubert. 2018. “Influence of the Geography of Speciation on Current Patterns of Coral Reef Fish Biodiversity across the Indo-Pacific.” *Ecography* 41 (8): 1295–1306.](http://paperpile.com/b/uQmu7a/1675)

[Goetze, Erica. 2005. “Global Population Genetic Structure and Biogeography of the Oceanic Copepods Eucalanus Hyalinus and E. Spinifer.” *Evolution; International Journal of Organic Evolution* 59 (11): 2378–98.](http://paperpile.com/b/uQmu7a/CUIP)

[———. 2011. “Population Differentiation in the Open Sea: Insights from the Pelagic Copepod Pleuromamma Xiphias.” *Integrative and Comparative Biology* 51 (4): 580–97.](http://paperpile.com/b/uQmu7a/Kv4i)

[Goudet, Jerome. 2005. “Hierfstat, a Package for R to Compute and Test Hierarchical F-Statistics.” *Molecular Ecology Notes* 5 (1): 184–86.](http://paperpile.com/b/uQmu7a/6CJA)

[Grorud-Colvert, Kirsten, and Su Sponaugle. 2011. “Variability in Water Temperature Affects Trait-Mediated Survival of a Newly Settled Coral Reef Fish.” *Oecologia* 165 (3): 675–86.](http://paperpile.com/b/uQmu7a/rFpw)

[Hodge, Jennifer R., and David R. Bellwood. 2016. “The Geography of Speciation in Coral Reef Fishes: The Relative Importance of Biogeographical Barriers in Separating Sister-Species.” *Journal of Biogeography* 43 (7): 1324–35.](http://paperpile.com/b/uQmu7a/1WaU)

[Holt, Ben G., Felix G. Marx, Susanne A. Fritz, Jean-Philippe Lessard, and Carsten Rahbek. 2020. “Evolutionary Diversification in the Marine Realm: A Global Case Study with Marine Mammals.” *Frontiers of Biogeography*. https://doi.org/](http://paperpile.com/b/uQmu7a/zsvL)[10.21425/f5fbg45184](http://dx.doi.org/10.21425/f5fbg45184)[.](http://paperpile.com/b/uQmu7a/zsvL)

[Irwin, Darren E. 2002. “Phylogeographic Breaks without Geographic Barriers to Gene Flow.” *Evolution; International Journal of Organic Evolution* 56 (12): 2383–94.](http://paperpile.com/b/uQmu7a/dom1)

[Jombart, Thibaut. 2008. “Adegenet: A R Package for the Multivariate Analysis of Genetic Markers.” *Bioinformatics*  24 (11): 1403–5.](http://paperpile.com/b/uQmu7a/cGOv)

[Laakkonen, Hanna M., Michael Hardman, Petr Strelkov, and Risto Väinölä. 2021. “Cycles of Trans-Arctic Dispersal and Vicariance, and Diversification of the Amphi-Boreal Marine Fauna.” *Journal of Evolutionary Biology* 34 (1): 73–96.](http://paperpile.com/b/uQmu7a/4smF)

[Lee, Taehwan, and Diarmaid O. Foighil. 2005. “Placing the Floridian Marine Genetic Disjunction into a Regional Evolutionary Context Using the Scorched Mussel, Brachidontes Exustus, Species Complex.” *Evolution; International Journal of Organic Evolution* 59 (10): 2139–58.](http://paperpile.com/b/uQmu7a/odG3)

[Momigliano, Paolo, Henri Jokinen, Antoine Fraimout, Ann-Britt Florin, Alf Norkko, and Juha Merilä. 2017. “Extraordinarily Rapid Speciation in a Marine Fish.” *Proceedings of the National Academy of Sciences of the United States of America* 114 (23): 6074–79.](http://paperpile.com/b/uQmu7a/NYc1)

[Neigel, J. E., and J. C. Avise. 1993. “Application of a Random Walk Model to Geographic Distributions of Animal Mitochondrial DNA Variation.” *Genetics* 135 (4): 1209–20.](http://paperpile.com/b/uQmu7a/2hqz)

[Nei, Masatoshi. 1987. “Molecular Evolutionary Genetics.” https://doi.org/](http://paperpile.com/b/uQmu7a/UrKS)[10.7312/nei-92038](http://dx.doi.org/10.7312/nei-92038)[.](http://paperpile.com/b/uQmu7a/UrKS)

[Nosil, P. 2012. “Ecological Speciation.”](http://paperpile.com/b/uQmu7a/ruqd) <https://books.google.com/books?hl=en&lr=&id=KygUDAAAQBAJ&oi=fnd&pg=PP1&dq=nosil+ecological+speciation&ots=s2HTRhOplV&sig=_qawenbGeay-sdPHSoLPLvA0aWU>[.](http://paperpile.com/b/uQmu7a/ruqd)

[Palumbi, S. R. 1992. “Marine Speciation on a Small Planet.” *Trends in Ecology & Evolution* 7 (4): 114–18.](http://paperpile.com/b/uQmu7a/8ivf)

[———. 1994. “Genetic Divergence, Reproductive Isolation, and Marine Speciation.” *Annual Review of Ecology and Systematics* 25 (1): 547–72.](http://paperpile.com/b/uQmu7a/4Lit)

[Potkamp, Gerrit, and Charles H. J. Fransen. 2019. “Speciation with Gene Flow in Marine Systems.” *Contributions to Zoology*  88 (2): 133–72.](http://paperpile.com/b/uQmu7a/c0GM)

[Prada, Carlos, and Michael E. Hellberg. 2020. “Speciation-by-Depth on Coral Reefs: Sympatric Divergence with Gene Flow or Cryptic Transient Isolation?” *Journal of Evolutionary Biology*, no. jeb.13731 (November). https://doi.org/](http://paperpile.com/b/uQmu7a/syGu)[10.1111/jeb.13731](http://dx.doi.org/10.1111/jeb.13731)[.](http://paperpile.com/b/uQmu7a/syGu)

[Robertson, D. Ross, and Katie L. Cramer. 2014. “Defining and Dividing the Greater Caribbean: Insights from the Biogeography of Shorefishes.” *PloS One* 9 (7): e102918.](http://paperpile.com/b/uQmu7a/9v6y)

[Robertson, D. Ross, Frances Karg, Rodrigo Leao de Moura, Benjamin C. Victor, and Giacomo Bernardi. 2006. “Mechanisms of Speciation and Faunal Enrichment in Atlantic Parrotfishes.” *Molecular Phylogenetics and Evolution* 40 (3): 795–807.](http://paperpile.com/b/uQmu7a/aAEN)

[Rocha, Luiz A., D. Ross Robertson, Joe Roman, and Brian W. Bowen. 2005. “Ecological Speciation in Tropical Reef Fishes.” *Proceedings. Biological Sciences / The Royal Society* 272 (1563): 573–79.](http://paperpile.com/b/uQmu7a/wd50)

[Searcy, Steven P., and Su Sponaugle. 2001. “Selective Mortality during the Larval–juvenile Transition in Two Coral Reef Fishes.” *Ecology* 82 (9): 2452–70.](http://paperpile.com/b/uQmu7a/Gz9A)

[Selkoe, Kimberly A., Steven D. Gaines, Jennifer E. Caselle, and Robert R. Warner. 2006. “Current Shifts and Kin Aggregation Explain Genetic Patchiness in Fish Recruits.” *Ecology* 87 (12): 3082–94.](http://paperpile.com/b/uQmu7a/yGq4)

[Taylor, Michael S., and Michael E. Hellberg. 2005. “Marine Radiations at Small Geographic Scales: Speciation in Neotropical Reef Gobies (Elacatinus).” *Evolution*. https://doi.org/](http://paperpile.com/b/uQmu7a/sHVL)[10.1554/04-590](http://dx.doi.org/10.1554/04-590)[.](http://paperpile.com/b/uQmu7a/sHVL)

[———. 2006. “Comparative Phylogeography in a Genus of Coral Reef Fishes: Biogeographic and Genetic Concordance in the Caribbean.” *Molecular Ecology* 15 (3): 695–707.](http://paperpile.com/b/uQmu7a/RwZp)

[Teske, Peter R., Jonathan Sandoval-Castillo, Tirupathi Rao Golla, Arsalan Emami-Khoyi, Mbaye Tine, Sophie von der Heyden, and Luciano B. Beheregaray. 2019. “Thermal Selection as a Driver of Marine Ecological Speciation.” *Proceedings. Biological Sciences / The Royal Society* 286 (1896): 20182023.](http://paperpile.com/b/uQmu7a/Rllt)

[Thompson, Julie D., Toby J. Gibson, and Des G. Higgins. 2002. “Multiple Sequence Alignment Using ClustalW and ClustalX.” *Current Protocols in Bioinformatics / Editoral Board, Andreas D. Baxevanis ... [et Al.]* Chapter 2 (August): Unit 2.3.](http://paperpile.com/b/uQmu7a/W25j)

[Wainwright, Peter C., Francesco Santini, David R. Bellwood, D. Ross Robertson, Luiz A. Rocha, and Michael E. Alfaro. 2018. “Phylogenetics and Geography of Speciation in New World Halichoeres Wrasses.” *Molecular Phylogenetics and Evolution* 121 (April): 35–45.](http://paperpile.com/b/uQmu7a/A7wY)

[Whitney, Jonathan L., Megan J. Donahue, and Stephen A. Karl. 2018. “Niche Divergence along a Fine‐scale Ecological Gradient in Sympatric Color Morphs of a Coral Reef Fish.” *Ecosphere*  9 (1): e02015.](http://paperpile.com/b/uQmu7a/ciwH)
