# Supplementary material for: Reevaluating claims of ecological speciation in Halichoeres bivittatus
Source: Ecol Evol. 2021 Jul 26;11(16):11449–56. doi: 10.1002/ece3.7936 (PMC8366890; doi:10.1002/ece3.7936)
Supplement: Supplementary file 2 — Supplementary Material [file ECE3-11-11449-s001.pdf]

Halichoeres Bivitattus Biogeography

Setup

```
library(babette)
library(mcbette)
library(beastier)
library(ape)
library(knitr)
library(phytools)
library(geiger)
library(DT)
library(sf)
library(leaflet)
library(tidyr)
library(plyr)
library(dplyr)
library(leaflet.minicharts)
library(ggplot2)
library(reshape2)
library(adegenet)
library(hierfstat)

setwd("~/Dropbox/Ongoing Projects/Hal Biv biogeography/")
```

Introduction

In this analysis we're going to take an alignment of cytochrome B from a bunch of *Halichoeres bivittatus* specimens, build a tree, and ask whether there's any indication that haplotypes are segregated by habitat in the Florida Keys. The first part of this analysis is very heavily based on this ROpenSci post by Richèl J.C. Bilderbeek

Alignment

First we'll load in our alignment and visualize it to make sure there aren't any obvious problems.

```
save_nexus_as_fasta("Halicheoeres NEW cytb_no_Out.nex",
                    "halbiv.fasta")

par0 <- par(mar = c(3, 7, 3, 1))
dna_sequences <- read.FASTA("halbiv.fasta")
image.DNAbin(dna_sequences, mar = c(3, 7, 3, 1))
```

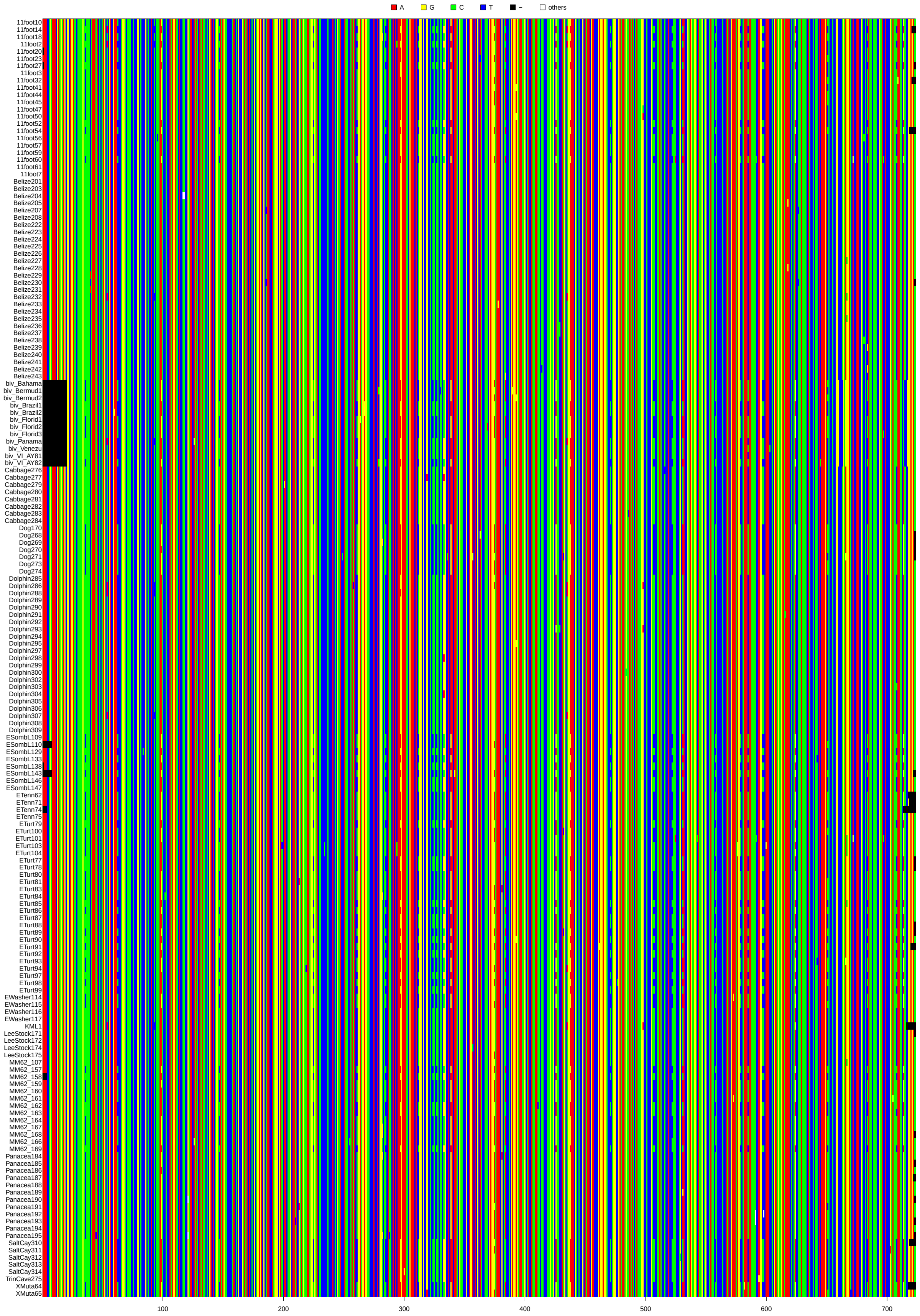
A
G
C
T
−
others
11foot10
11foot14
11foot18
11foot2
11foot20
11foot23
11foot27
11foot3
11foot32
11foot41
11foot44
11foot45
11foot47
11foot50
11foot52
11foot54
11foot56
11foot57
11foot59
11foot60
11foot61
11foot7
Belize201
Belize203
Belize204
Belize205
Belize207
Belize208
Belize222
Belize223
Belize224
Belize225
Belize226
Belize227
Belize228
Belize229
Belize230
Belize231
Belize232
Belize233
Belize234
Belize235
Belize236
Belize237
Belize238
Belize239
Belize240
Belize241
Belize242
Belize243
biv_Bahama
biv_Bermud1
biv_Bermud2
biv_Brazil1
biv_Brazil2
biv_Florid1
biv_Florid2
biv_Florid3
biv_Panama
biv_Venezu
biv_VI_AY81
biv_VI_AY82
Cabbage276
Cabbage277
Cabbage279
Cabbage280
Cabbage281
Cabbage282
Cabbage283
Cabbage284
Dog170
Dog268
Dog269
Dog270
Dog271
Dog273
Dog274
Dolphin285
Dolphin286
Dolphin288
Dolphin289
Dolphin290
Dolphin291
Dolphin292
Dolphin293
Dolphin294
Dolphin295
Dolphin297
Dolphin298
Dolphin299
Dolphin300
Dolphin302
Dolphin303
Dolphin304
Dolphin305
Dolphin306
Dolphin307
Dolphin308
Dolphin309
ESombL109
ESombL110
ESombL129
ESombL133
ESombL138
ESombL143
ESombL146
ESombL147
ETenn62
ETenn71
ETenn74
ETenn75
ETurt79
ETurt100
ETurt101
ETurt103
ETurt104
ETurt77
ETurt78
ETurt80
ETurt81
ETurt83
ETurt84
ETurt85
ETurt86
ETurt87
ETurt88
ETurt89
ETurt90
ETurt91
ETurt92
ETurt93
ETurt94
ETurt97
ETurt98
ETurt99
EWasher114
EWasher115
EWasher116
EWasher117
KML1
LeeStock171
LeeStock172
LeeStock174
LeeStock175
MM62_107
MM62_157
MM62_158
MM62_159
MM62_160
MM62_161
MM62_162
MM62_163
MM62_164
MM62_167
MM62_168
MM62_166
MM62_169
Panacea184
Panacea185
Panacea186
Panacea187
Panacea188
Panacea189
Panacea190
Panacea191
Panacea192
Panacea193
Panacea194
Panacea195
SaltCay310
SaltCay311
SaltCay312
SaltCay313
SaltCay314
TrinCave275
XMuta64
XMuta65
100
200
300
400
500
600
700

Building the tree

Note This is the inference model for the tn93 analysis, the full Bayesian one was done by Ron Eytan manually.

```
inf.model <- create_inference_model(
  site_model = create_tn93_site_model()
)
trees <- bbt_run_from_model("halbiv.fasta",
                            inference_model = inf.model)
save(trees, file = "halbiv-beast.Rda")
contree <- consensus(trees$halbiv_trees[1001:10001])
save(contree, file = "contree.Rda")
```

```
trees <- read.nexus("./HalichoeresNEWcytb_no_OutStrictCLKbMTnoCalibration.trees")
save(trees, file = "halbiv-beast.Rda")
contree <- consensus(trees[seq(1001, 50001, by = 10)])
save(contree, file = "contree.Rda")
```

Densitree plot

```
load("halbiv-beast.Rda")
load("contree.Rda")

plot_densitree(
  trees[seq(1001, 50001, by = 1000)],
  alpha = 0.1
)
```

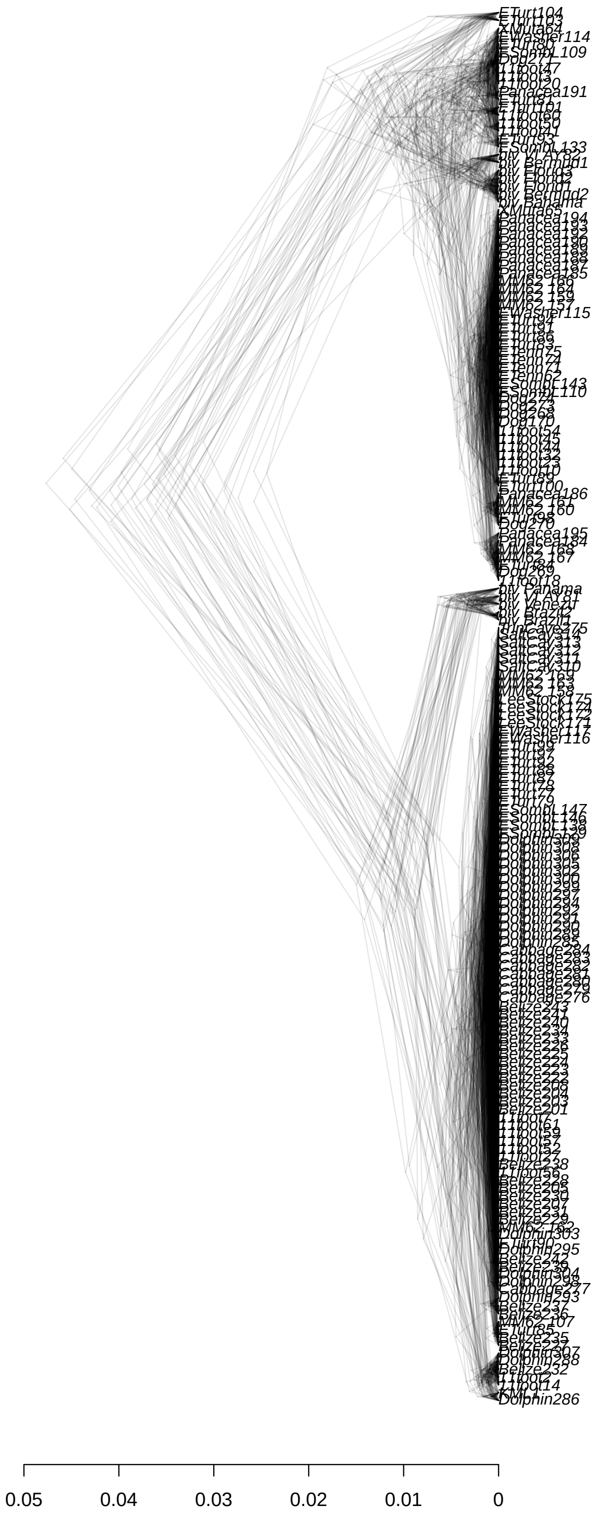
ETurt104
Panacea191
Panacea186
Dolphin286
0.05
0.04
0.03
0.02
0.01
0

```r
pdf("densitree.pdf", height = 34)
plot_densitree(
  trees[seq(1001, 50001, by = 1000)],
  alpha = 0.1
)
dev.off()
```

```
## pdf
##   2
```

As expected based on previous analyses and the Rocha et al. results, there's a very deep split here that is very highly supported. There's little resolution at shallow nodes, but the north/south split is very strong. The lineage at the top constitutes the "northern" haplotype, and includes the specimens from northern Florida (Dog Island, Panacea, St. Petersburg) as well as some of the specimens from the Bahamas and Bermuda. It also includes about half of the specimens from the Keys. The southern split, which also contains about half of the Keys specimens, also contains the specimens from Venezuela, the Virgin Islands, Panama, Brazil, some of the Bahamas specimens, and Belize.

Thus at the broad geographic scale the overall story here is the same as in Rocha et al.; a very deep split between a northern and southern haplotype, with a mix of the two at a suture zone in the Keys and the Bahamas. Rocha et al. said there's also a mix in Bermuda, but we don't have the data to address that issue here.

Strict consensus tree

In order to condense this down to N/S haplotype assignments, we'll take a strict consensus tree from that MCMC run.

```r
plot(contree, type = "radial", lab4ut = "axial")
nodelabels(c("Southern", "Northern"), node = c(179, 183))
```

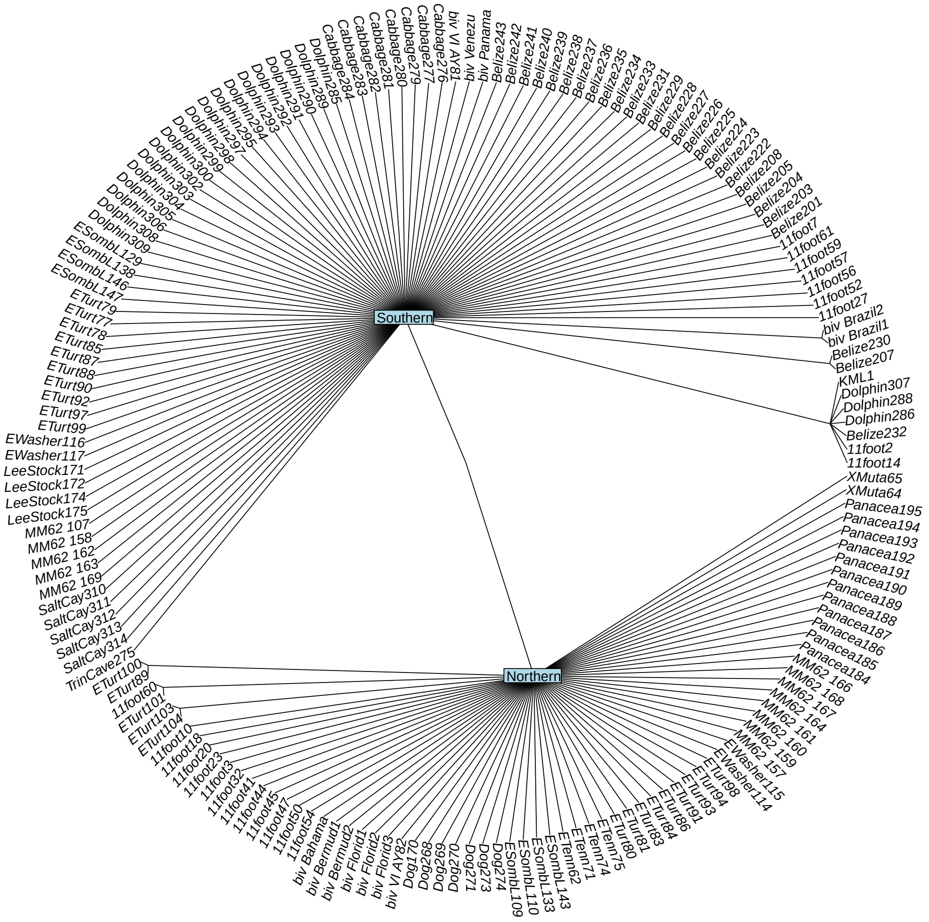


```r
pdf("contree.pdf", width = 15, height = 15)
plot(contree, type = "radial", lab4ut = "axial")
nodelabels(c("Southern", "Northern"), node = c(179, 183))
dev.off()
```

```
## pdf
##   2
```

From examining the tree we can see that the southern haplotype is represented by the clade descending from node 179 and the northern haplotype is node 184.

Extracting clade membership

```
habitat.df <- read.csv("habitat by specimen.csv")
habitat.df$haplotype <- rep(NA, nrow(habitat.df))
habitat.df[habitat.df$Specimen %in% tips(contree, 179),"haplotype"] <- "S"
habitat.df[habitat.df$Specimen %in% tips(contree, 183),"haplotype"] <- "N"

datatable(habitat.df, extensions = 'Buttons',
          options = list(dom = 'Blfrtip',
                         buttons = c('copy', 'csv',
                                     'excel', 'pdf', 'print'),
                         columnDefs = list(list(className = 'dt-center',
                                                targets = 0:ncol(habitat.df))),
                         lengthMenu = list(c(10,25,50,-1),
                                           c(10,25,50,"All"))))
```

Copy CSV PDF Print Show 10 entries Search:

	Specimen	Habitat.type	Location	Site	haplotype
1	11foot10	Reef	Offshore	11 Foot Mound	N
2	11foot14	Reef	Offshore	11 Foot Mound	S
3	11foot18	Reef	Offshore	11 Foot Mound	N
4	11foot2	Reef	Offshore	11 Foot Mound	S
5	11foot20	Reef	Offshore	11 Foot Mound	N
6	11foot23	Reef	Offshore	11 Foot Mound	N
7	11foot27	Reef	Offshore	11 Foot Mound	S
8	11foot3	Reef	Offshore	11 Foot Mound	N
9	11foot32	Reef	Offshore	11 Foot Mound	N
10	11foot41	Reef	Offshore	11 Foot Mound	N

Showing 1 to 10 of 177 entries Previous 1 2 3 4 5 … 18 Next

Data aggregation

```
localities <- read_sf("./doc.kml") %>%
  as_Spatial() %>%
  as.data.frame() %>%
  select(Name, coords.x1, coords.x2) %>%
  filter(!Name == "East East Turtle Shoal")

colnames(localities) <- c("Site", "Longitude", "Latitude")

hapcount.df <- habitat.df %>%
```

```
  na.omit() %>%
  dplyr::count(Site, Habitat.type, Location, haplotype) %>%
  spread(key = haplotype, value = n, fill = 0)

localities <- join(localities, hapcount.df, by = "Site")
```

Plotting sampling locations

```
datatable(localities, extensions = 'Buttons',
          options = list(dom = 'Blfrtip',
                         buttons = c('copy', 'csv',
                                     'excel', 'pdf', 'print'),
                         columnDefs = list(list(className = 'dt-center',
                                               targets = 0:ncol(localities))),
                         lengthMenu = list(c(10,25,50,-1),
                                           c(10,25,50,"All")))) %>%
  formatRound(columns=c("Longitude", "Latitude"), digits=3)
```

Copy CSV PDF Print Show 10 entries Search:

	Site	Longitude	Latitude	Habitat.type	Location	N	S
1	11 Foot Mound	-80.862	24.723	Reef	Offshore	13	9
2	East side of Tennessee Reef	-80.778	24.746	Reef	Offshore	4	0
3	KML	-80.815	24.826	Grass	Inshore	0	1
4	XMuta	-80.687	24.800	Reef	Offshore	2	0
5	East Turtle Shoal	-80.930	24.721	Reef	Inshore	14	10
6	MM62	-80.904	24.784	Grass	Inshore	8	5
7	East of Sombrero Light	-81.103	24.628	Reef	Offshore	4	4
8	East Washerwoman	-81.072	24.665	Reef	Inshore	2	2

Showing 1 to 8 of 8 entries Previous 1 Next

Haplotype frequencies by sampling site

Since we don't have the non-Florida samples in the KMZ file, I'm going to add them manually for plotting purposes. In the sampling file we have a bunch of samples labled "Dolphin Encounters" and some others labels "Salt Cay". The "Dolphin Encounters" samples were collected by DW and RE, and I think the "Salt Cay" ones were collected by PW. Dolphin Encounters is actually on Salt Cay, though, so we're going to merge those together for plotting purposes.

Note that some of these are based on samples posted to Genbank by Rocha et al., and those only have country names associated with them, not latitudes and longitudes. Placement of those pie charts is therefore approximate.

```
colors <- c("#f5dc7a", "#a532ed")

all.localities <- read.csv("all.localities.csv")

leaflet(all.localities) %>%
  addProviderTiles(provider = "GeoportailFrance.orthos") %>%
  addCircleMarkers(label = ~ Site, opacity = 0, fillOpacity = 0,
                   radius = 20) %>%
  addMinicharts(all.localities$Longitude, all.localities$Latitude,
                type = "pie",
                chartdata = all.localities[,c("N", "S")],
                colorPalette = colors)
```

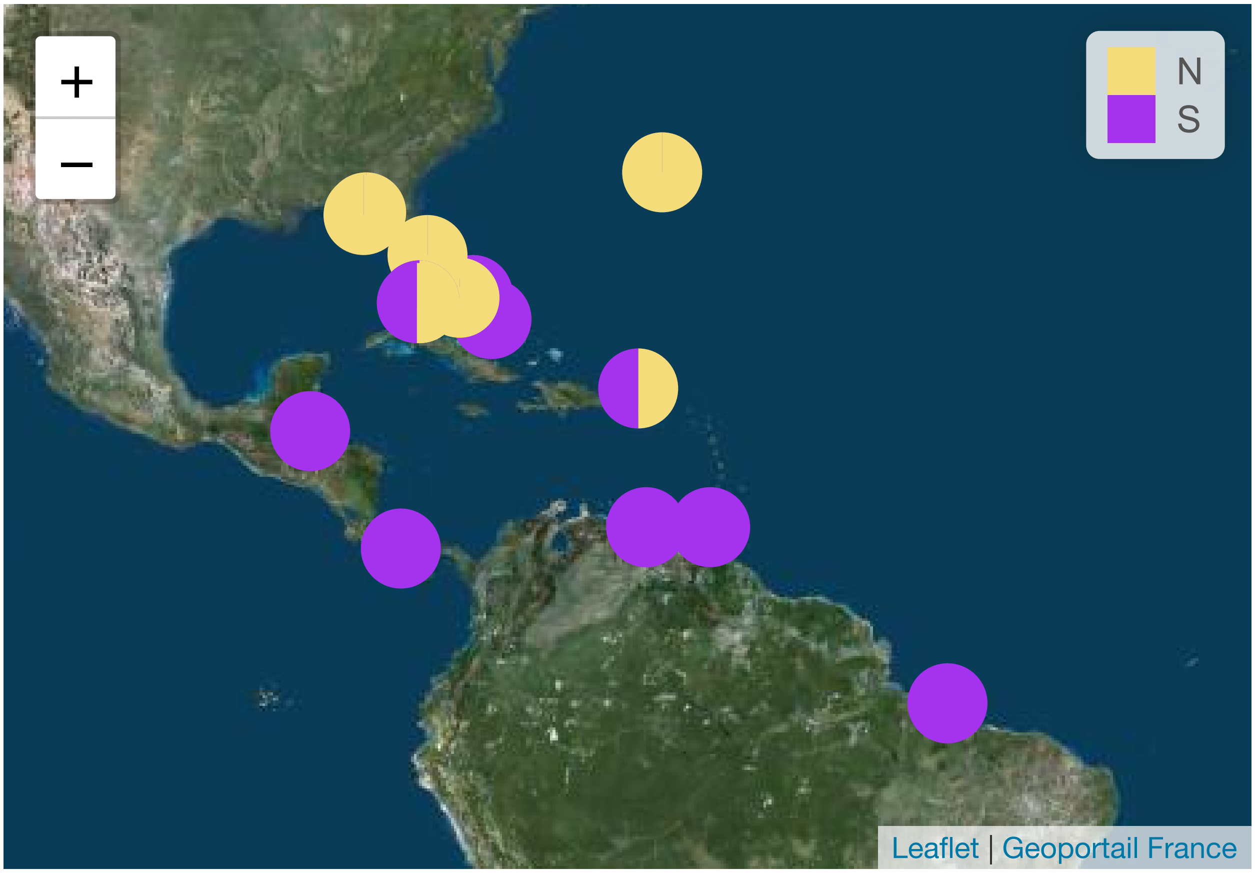

Habitat segregation in the Florida Keys

Haplotpye frequencies, Keys only

```
colors <- c("#f5dc7a", "#a532ed")
```

```r
leaflet(localities) %>%
  addProviderTiles(provider = "GeoportailFrance.orthos") %>%
  addCircleMarkers(label = ~ Site, opacity = 0, fillOpacity = 0,
                   radius = 20) %>%
  addMinicharts(localities$Longitude, localities$Latitude,
                type = "pie",
                chartdata = localities[,c("N", "S")],
                colorPalette = colors)
```

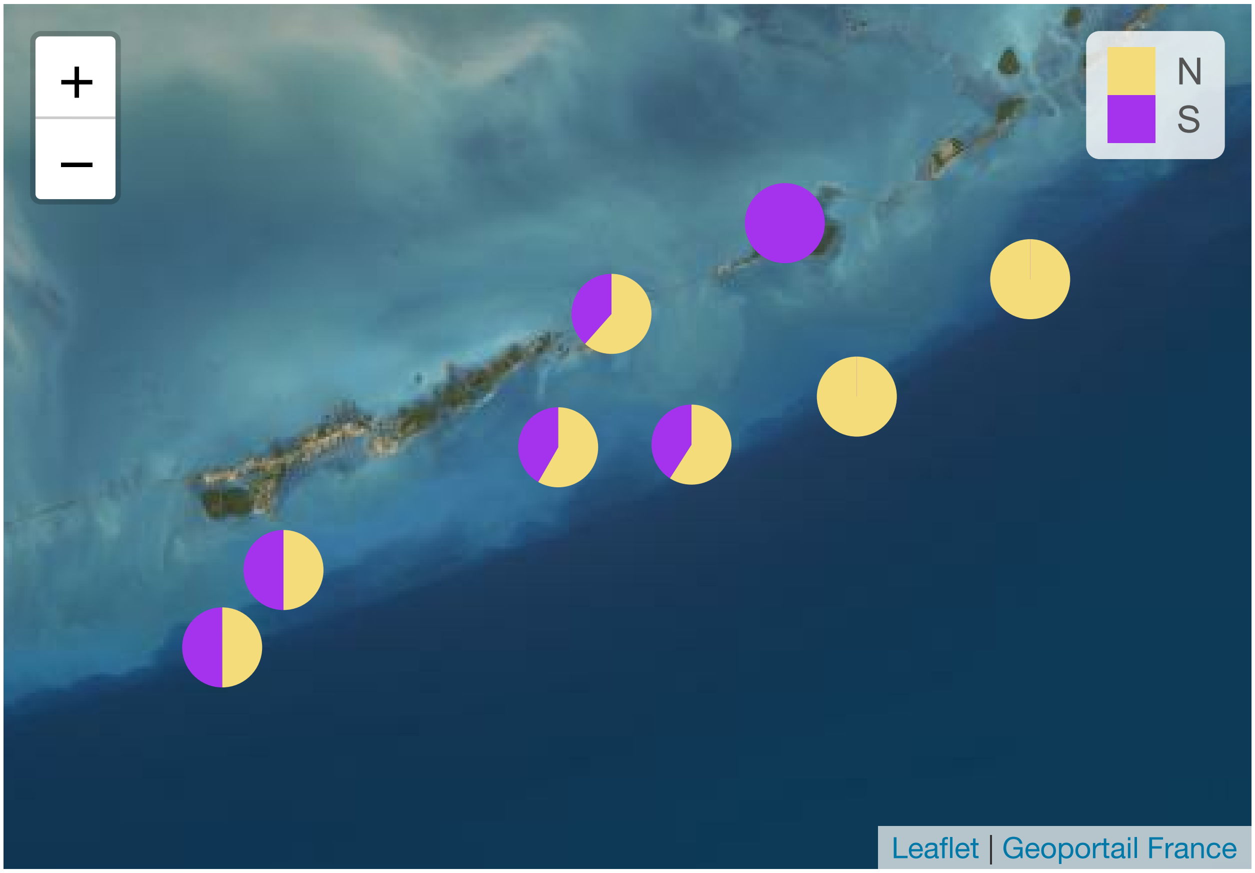

Here we see that both haplotypes are found at somehwat high frequencies at most sites. The two exceptions (KML, X Muta, and the east side of Tennessee Reef) are the two with the smallest sample sizes (1, 2, and 4 samples, respectively). As a result it's probably best not to read too much into those sites.

This next bit of code is just re-making the pie charts so we can assemble a vector map in Illustrator.

```r
all.localities.long <- melt(all.localities,
                            id.vars = c("Site", "Longitude", "Latitude", "Habitat.type", "Location"),
                            variable.name = "Haplotype")

for(i in unique(all.localities.long$Site)){
  this.df <- all.localities.long %>%
    filter(Site == i)

  pdf(file = paste0(i, " pie chart.pdf"))
  print(ggplot(this.df, aes(x="", y=value, fill=Haplotype)) +
    geom_bar(stat="identity", width=2, color = "black") +
```

```
    coord_polar("y", start=0) + theme_void() +
    scale_fill_manual(values = c("#404040", "#D0D0D0")))
  dev.off()
}
```

By habitat type

Haplotype frequencies by habitat type

```
localities %>%
  pivot_longer(c("N", "S"), names_to = "haplotype", values_to = "count") %>%
  ggplot(aes(fill=haplotype, y=count, x=Habitat.type)) +
  geom_bar(position="fill", stat="identity")
```

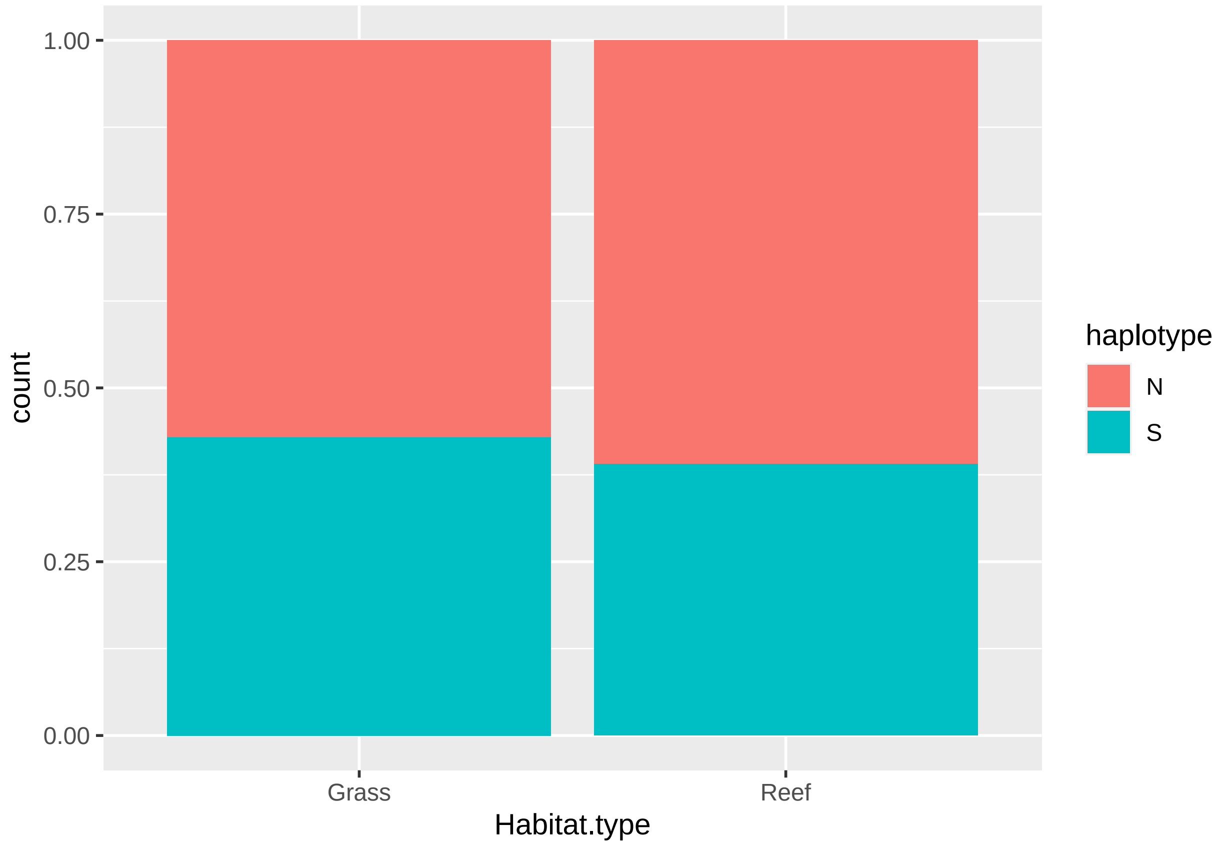


```
pdf("freq.by.type.pdf")
localities %>%
  pivot_longer(c("N", "S"), names_to = "haplotype", values_to = "count") %>%
  ggplot(aes(fill=haplotype, y=count, x=Habitat.type)) +
  geom_bar(position="fill", stat="identity")
dev.off()
```

```
## pdf
##   2
```

In this plot we're comparing haplotype frequencies for shallow (< 2m) grass bed communities to those for reef communities, combining both patch reef and fore reef. Again we see very similar frequencies.

Genetic distance between habitat types

We're going to measure Fst between all habitat types in the Florida Keys, and then compare those values to the distribution of pairwise Fsts we get from permutation tests. For these permutations, we simply randomly permute the population vector and then measure Fsts again, giving us a distribution of expected pairwise Fsts under the hypothesis that there is no habitat segregation. We're doing 1000 permutations.

```
fl.sequences <- subset(dna_sequences, names(dna_sequences) %in%
                         habitat.df[complete.cases(habitat.df),"Specimen"])
halbiv.genind <- DNAbin2genind(fl.sequences)
fl.specimens <- rownames(halbiv.genind$tab)
fl.habitat.df <- habitat.df[habitat.df$Specimen %in% fl.specimens,]
rownames(fl.habitat.df) <- fl.habitat.df$Specimen
fl.habitat.df$full.habitat <- paste(fl.habitat.df$Location,
                                    fl.habitat.df$Habitat.type)

halbiv.genind$pop <- as.factor(fl.habitat.df[fl.specimens, "full.habitat"])

fst.dist <- as.matrix(genet.dist(halbiv.genind, method="Nei87"))

if(file.exists("habitat.fst.rank.csv")){
  habitat.rank.df <- read.csv("habitat.fst.rank.csv", row.names = 1)
  colnames(habitat.rank.df) <- rownames(habitat.rank.df)
} else {

  sims <- list()
  nsims <- 1000

  for(i in 1:nsims){
    this.genind <- halbiv.genind
    this.genind$pop <- sample(this.genind$pop)
    sims[[i]] <- as.matrix(genet.dist(this.genind, method="Nei87"))
  }

  # Step through each row/column combo and figure out the
  # ranking of our empirical result in the simulations.

  habitat.rank.df <- as.data.frame(fst.dist)
  habitat.rank.df[,] <- NA

  for(i in 1:nrow(fst.dist)){
    for(j in 1:ncol(fst.dist)){
      habitat.rank.df[i,j] <- rank(c(fst.dist[i,j], sapply(sims, function(x) x[i,j])))[1]
    }
  }

  habitat.rank.df <- 1 - habitat.rank.df/(nsims + 1)

  write.csv(habitat.rank.df, file = "habitat.fst.rank.csv")
}
```

```r
datatable(habitat.rank.df, extensions = 'Buttons',
          options = list(dom = 'Blfrtip',
                         buttons = c('copy', 'csv',
                                     'excel', 'pdf', 'print'),
                         columnDefs = list(list(className = 'dt-center',
                                                targets = 0:ncol(habitat.rank.df))),
                         lengthMenu = list(c(10,25,50,-1),
                                           c(10,25,50,"All")))) %>%
  formatRound(columns=colnames(habitat.rank.df), digits=3)
```

Copy CSV PDF Print Show 10 entries Search:

	Inshore Grass	Inshore Reef	Offshore Reef
Inshore Grass	0.500	0.829	0.642
Inshore Reef	0.829	0.500	0.494
Offshore Reef	0.642	0.494	0.500

Showing 1 to 3 of 3 entries Previous 1 Next

```r
melted.cor.mat <- reshape2::melt(as.matrix(habitat.rank.df))
colnames(melted.cor.mat) <- c("Site1", "Site2", "value")

cor.heatmap <- ggplot(data = melted.cor.mat, aes_string(x="Site1", y="Site2", fill="value")) +
  geom_tile() + scale_fill_viridis_c(direction = -1, limits = c(0,1)) +
  theme(axis.text.x = element_text(angle = 90, hjust = 1)) +
  theme(panel.grid.major = element_blank(), panel.grid.minor = element_blank(),
        panel.background = element_blank())
cor.heatmap
```

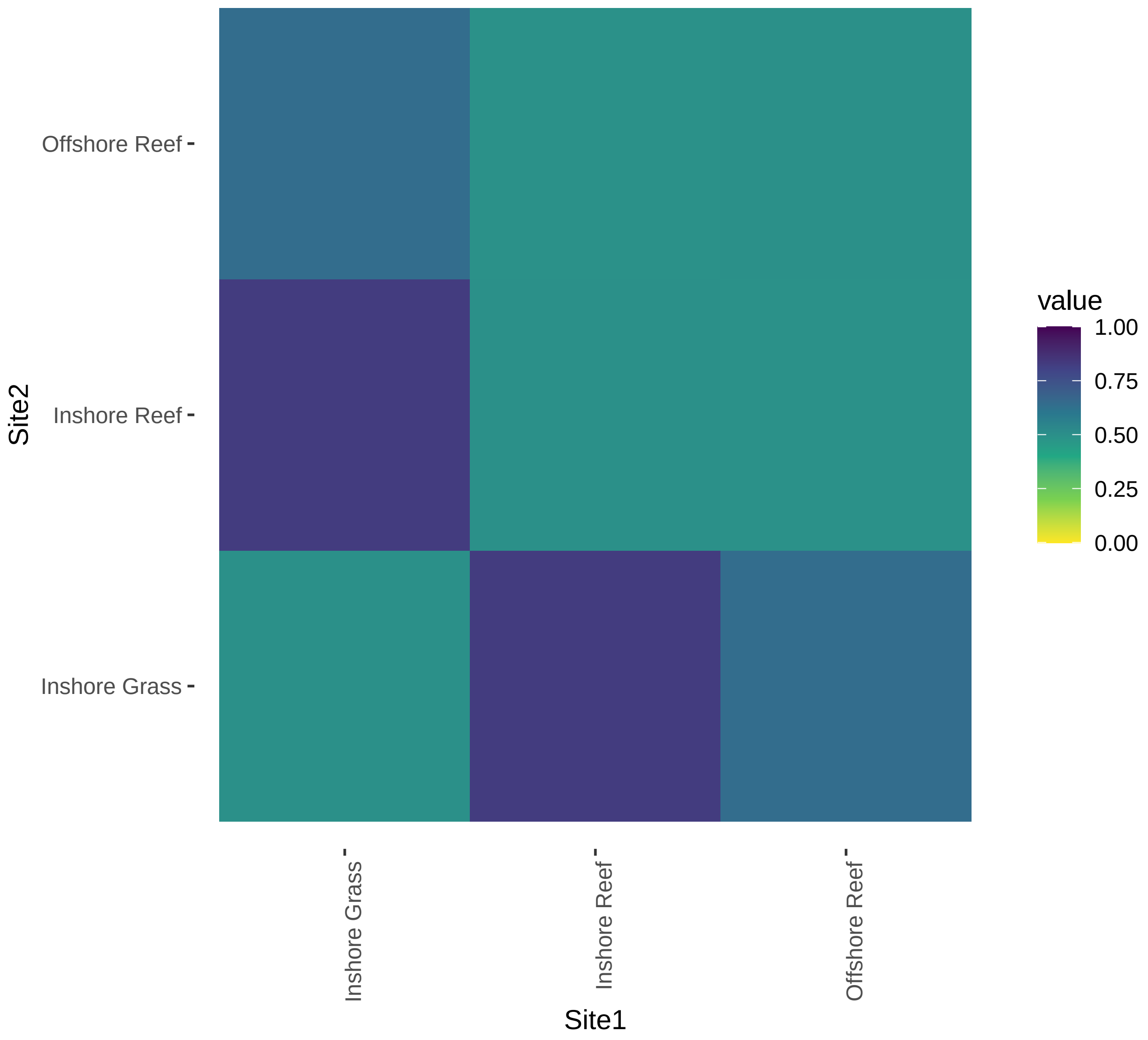


```
pdf("fst.by.type.pdf")
cor.heatmap
dev.off()
```

```
## pdf
##   2
```

By site

Haplotype frequencies by site category (onshore vs. offshore)

```
localities %>%
  pivot_longer(c("N", "S"), names_to = "haplotype", values_to = "count") %>%
  ggplot(aes(fill=haplotype, y=count, x=Location)) +
  geom_bar(position="fill", stat="identity")
```

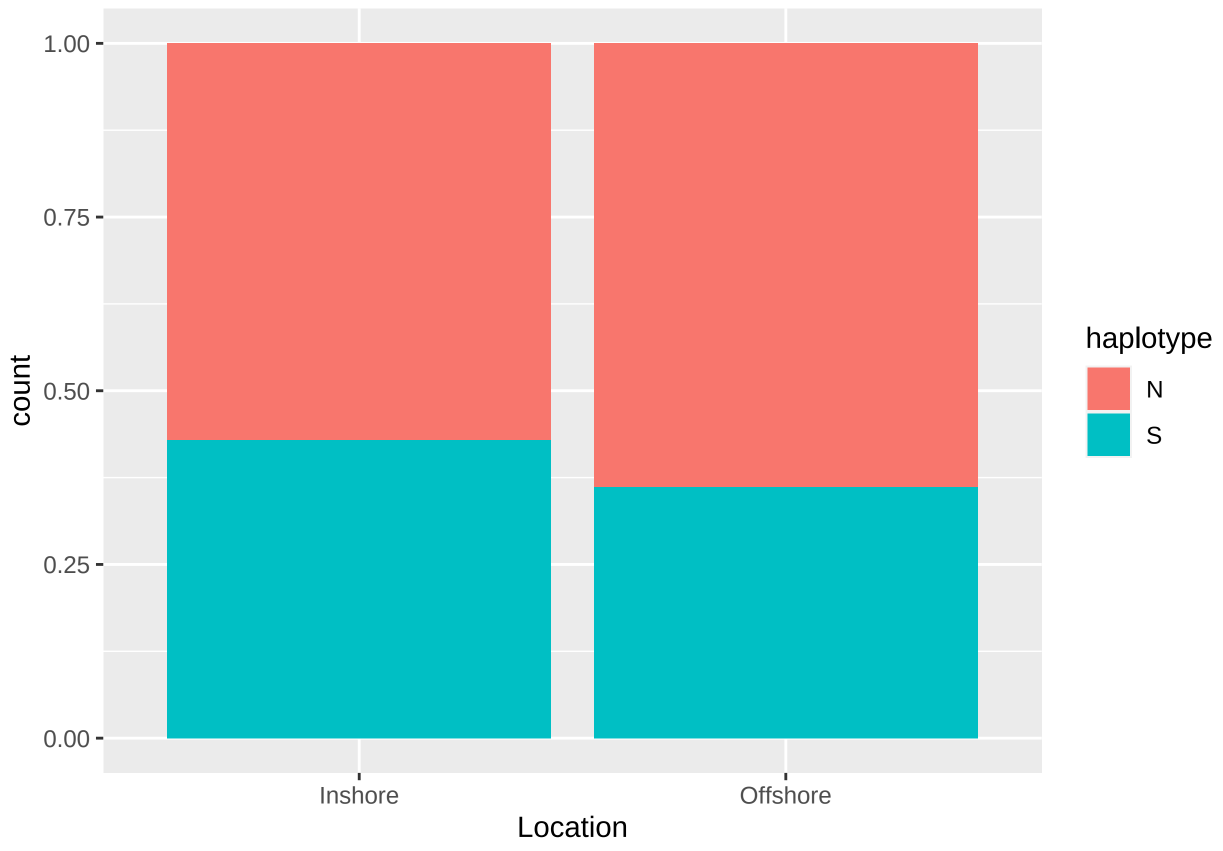


```
pdf("hap.freqs.by.site")
localities %>%
  pivot_longer(c("N", "S"), names_to = "haplotype", values_to = "count") %>%
  ggplot(aes(fill=haplotype, y=count, x=Location)) +
  geom_bar(position="fill", stat="identity")
dev.off()
```

```
## pdf
##   2
```

Here we see that the haplotype frequencies in onshore and offshore sites are not very different from one another, and to the extent that they do differ it is in the opposite direction predicted by the Rocha et al. (2015) hypothesis; we find slightly higher prevalence of the southern haplotype at the inshore patch reefs and grass bed sites, which are expected to experience greater fluctuations in temperature.

Genetic distance between sites

We're going to measure Fst between all sites in the Florida Keys, and then compare those values to the distribution of pairwise Fsts we get from permutation tests. For these permutations, we simply randomly permute the population vector and then measure Fsts again, giving us a distribution of expected pairwise Fsts under the hypothesis that there is no habitat segregation. We're doing 1000 permutations.

```
halbiv.genind <- DNAbin2genind(fl.sequences)
fl.specimens <- rownames(halbiv.genind$tab)
```

```r
fl.habitat.df <- habitat.df[habitat.df$Specimen %in% fl.specimens,]
rownames(fl.habitat.df) <- fl.habitat.df$Specimen

halbiv.genind$pop <- as.factor(fl.habitat.df[fl.specimens, "Site"])

fst.dist <- as.matrix(genet.dist(halbiv.genind, method="Nei87"))

if(file.exists("site.fst.rank.csv")){
  site.rank.df <- read.csv("site.fst.rank.csv", row.names = 1)
  colnames(site.rank.df) <- rownames(site.rank.df)
} else {

  sims <- list()
  nsims <- 1000

  for(i in 1:nsims){
    this.genind <- halbiv.genind
    this.genind$pop <- sample(this.genind$pop)
    sims[[i]] <- as.matrix(genet.dist(this.genind, method="Nei87"))
  }

  # Step through each row/column combo and figure out the
  # ranking of our empirical result in the simulations.

  site.rank.df <- as.data.frame(fst.dist)
  site.rank.df[,] <- NA

  for(i in 1:nrow(fst.dist)){
    for(j in 1:ncol(fst.dist)){
      site.rank.df[i,j] <- rank(c(fst.dist[i,j], sapply(sims, function(x) x[i,j])))[1]
    }
  }

  site.rank.df <- 1 - site.rank.df/(nsims + 1)

  write.csv(site.rank.df, file = "site.fst.rank.csv")
}

datatable(site.rank.df, extensions = 'Buttons',
          options = list(dom = 'Blfrtip',
                         buttons = c('copy', 'csv',
                                     'excel', 'pdf', 'print'),
                         columnDefs = list(list(className = 'dt-center',
                                                targets = 0:ncol(site.rank.df))),
                         lengthMenu = list(c(10,25,50,-1),
                                           c(10,25,50,"All")))) %>%
  formatRound(columns=colnames(site.rank.df), digits=3)
```

Copy CSV PDF Print Show 10 entries Search:

	11 Foot Mound	KML	East of Sombrero Light	East side of Tennessee Reef	East Turtle Shoal	East Washerwoman	MM62	XMuta
11 Foot Mound	0.500	0.145	0.768	0.088	0.839	0.904	0.719	0.324
KML	0.145	0.500	0.304	0.000	0.131	0.351	0.118	0.122
East of Sombrero Light	0.768	0.304	0.500	0.057	0.800	0.994	0.616	0.222
East side of Tennessee Reef	0.088	0.000	0.057	0.500	0.090	0.069	0.132	0.401
East Turtle Shoal	0.839	0.131	0.800	0.090	0.500	0.914	0.736	0.307
East Washerwoman	0.904	0.351	0.994	0.069	0.914	0.500	0.787	0.294
MM62	0.719	0.118	0.616	0.132	0.736	0.787	0.500	0.356
XMuta	0.324	0.122	0.222	0.401	0.307	0.294	0.356	0.500

Showing 1 to 8 of 8 entries Previous 1 Next

```
melted.cor.mat <- reshape2::melt(as.matrix(site.rank.df))
colnames(melted.cor.mat) <- c("Site1", "Site2", "value")

cor.heatmap <- ggplot(data = melted.cor.mat, aes_string(x="Site1", y="Site2", fill="value")) +
  geom_tile() + scale_fill_viridis_c(direction = -1, limits = c(0,1)) +
  theme(axis.text.x = element_text(angle = 90, hjust = 1)) +
  theme(panel.grid.major = element_blank(), panel.grid.minor = element_blank(),
        panel.background = element_blank())
cor.heatmap
```

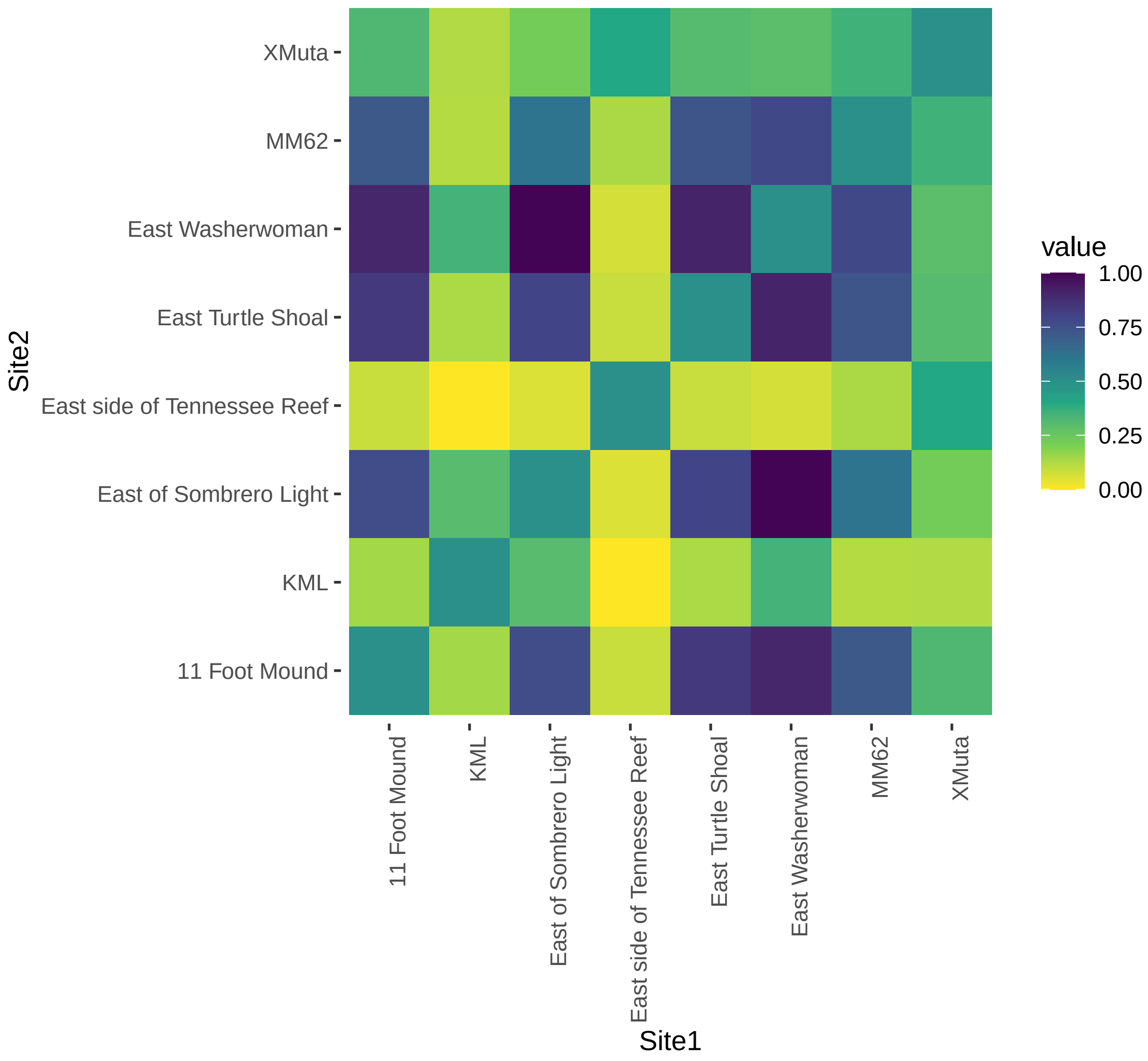


```r
pdf("fst.by.site.pdf")
cor.heatmap
dev.off()
```

```
## pdf
##   2
```

Just for simplicity's sake I'm going to set all p > .05 to 1 and re-plot that heatmap to highlight which comparisons are significant.

```r
melted.cor.mat[which(melted.cor.mat$value > .05), "value"] <- 1
cor.heatmap <- ggplot(data = melted.cor.mat, aes_string(x="Site1", y="Site2", fill="value")) +
  geom_tile() + scale_fill_viridis_c(direction = -1, limits = c(0,1)) +
  theme(axis.text.x = element_text(angle = 90, hjust = 1)) +
  theme(panel.grid.major = element_blank(), panel.grid.minor = element_blank(),
        panel.background = element_blank())
cor.heatmap
```

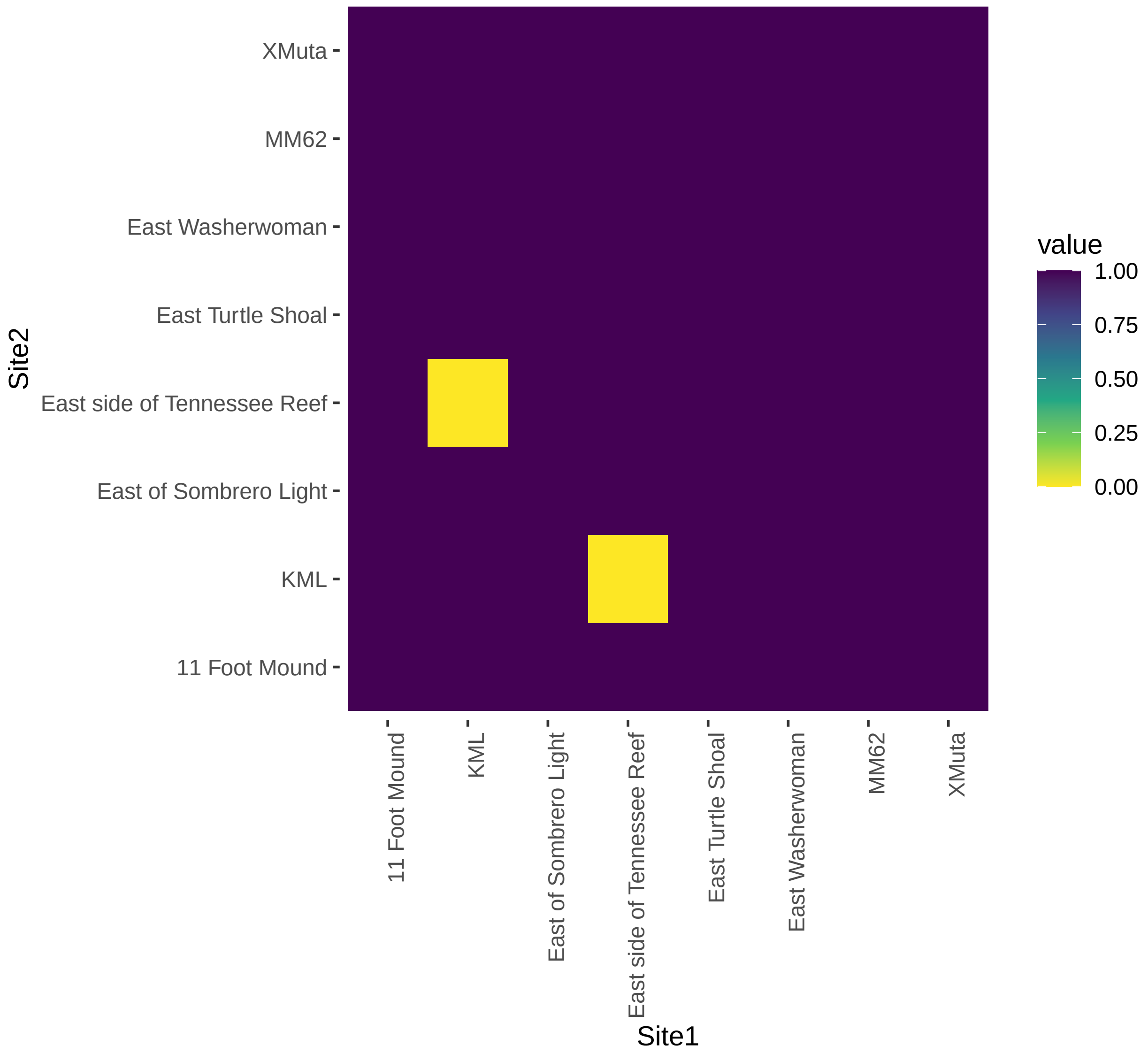


```
pdf("fst.by.site.sigonly.pdf")
cor.heatmap
dev.off()
```

```
## pdf
##   2
```

So the only comparison that is statistically significant (without Bonferroni correction) is the comparison between East Tennessee Reef and the grass bed behind KML. However the sample sizes are miniscule there (4 vs. 1), and the direction of difference seen is exactly opposite to that predicted by the Rocha et al. result, with the northern haplotype dominating out on the dropoff and the KML specimen being the southern haplotype.
